# Increased Red Cell Distribution Width Is Associated With Disease Severity in Hospitalized Adults With SARS-CoV-2 Infection: An Observational Multicentric Study

**DOI:** 10.3389/fmed.2020.616292

**Published:** 2020-12-11

**Authors:** Theodoros Karampitsakos, Karolina Akinosoglou, Ourania Papaioannou, Vassiliki Panou, Athanasios Koromilias, Petros Bakakos, Stelios Loukides, Demosthenes Bouros, Charalampos Gogos, Argyrios Tzouvelekis

**Affiliations:** ^1^Department of Respiratory Medicine, University Hospital of Patras, Patras, Greece; ^2^Department of Internal Medicine, University General Hospital of Patras, Patras, Greece; ^3^First Academic Department of Pneumonology, Hospital for Diseases of the Chest, “Sotiria,” Medical School, National and Kapodistrian University of Athens, Athens, Greece; ^4^Second Academic Department of Respiratory Medicine, ATTIKON General Hospital, National and Kapodistrian University of Athens, Athens, Greece

**Keywords:** SARS-CoV-2, COVID-19, biomarkers, mortality, red cell distribution width (RDW)

## Abstract

**Background:** There is an amenable need for clinically applicable biomarkers in patients with SARS-CoV-2 infection. Red Cell Distribution Width (RDW) has been recently suggested as a prognostic biomarker for COVID-19.

**Methods:** This was an observational study enrolling patients between February 26 and May 15 2020. We aimed to validate the association of the previously published RDW threshold of 14.5% with markers of disease progression and mortality.

**Results:** A total number of 193 hospitalized patients with COVID-19 were enrolled and analyzed. Median age was 61 years (95% CI: 58–64). Patients with baseline RDW ≥14.5% (*n* = 41, 19.2%) presented with more progressive disease compared to patients with baseline RDW <14.5% (*n* = 156, 80.8%) as indicated by significant differences in maximum FiO2% during hospitalization (median: 100, 95% CI: 45.2–100, vs. 35, 95% CI: 31–40, *p* = 0.0001, respectively). Values of RDW ≥14.5% were also strongly associated with increased risk of mortality (HR: 4.1, 95% CI: 0.88–19.23), (*p* = 0.02).

**Conclusion:** Our study provides evidence to support reproducibility and validity of a specified cut-off threshold of RDW as biomarker of disease severity and mortality in patients with COVID-19.

## Introduction

The role of red cell distribution width (RDW) as a prognostic biomarker in various chronic lung diseases has gained much of attention (1, 2). Recently, our study group demonstrated that increased RDW is associated with poor clinical outcomes in chronic lung diseases, including chronic obstructive pulmonary disease and idiopathic pulmonary fibrosis (2, 3). RDW reflects the variation of red blood cell volumes and represents a relatively reproducible biomarker, considering the relatively prolonged lifespan of red blood cells. Nonetheless, underlying mechanisms of RDW elevation remain to be addressed. It has been suggested that arterial hypoxemia leads to increased erythropoietin secretion and thus to increased RDW through mechanisms that involve regulation of erythrocyte maturation and survival (2). Studies have shown that in patients with Coronavirus Disease 2019 (COVID-19), the severity of hypoxemia is independently associated with in-hospital mortality and can reliably predict admission to the intensive care unit (4). Unfortunately, oxygen saturation is often dissociated from arterial hypoxemia as well as the sense of dyspnea in patients with COVID-19 (5, 6). To this end, additional determinants of hypoxemia that are minimally invasive and clinicians' friendly are urgently needed for patients with COVID-19 (7). RDW has been suggested, in combination with hemoglobin and neutrophil to lymphocyte ratio, as a diagnostic and prognostic biomarker of COVID-19 (7–10). Recently, a large prospective study by Foy et al. demonstrated that elevated baseline RDW (>14.5%) levels were independently associated with worse clinical outcomes in hospitalized patients with COVID-19 (11). Despite relative enthusiasm, peripheral blood biomarkers, including RDW, need to be validated and reproduced in multiple cohorts from different institutions to support their widespread clinical applicability. To this end, we aimed to further validate in a completely different cohort of patients with COVID-19 (validation cohort), derived from a low incidence and mortality country (Greece), the association of RDW threshold of 14.5%, previously published by Foy et al. (derivation cohort) with markers of disease progression and mortality (11).

## Methods

This was an observational, multicentric study enrolling patients from six reference centers for COVID-19 in Greece. From February 26 to May 15 2020 epidemiological and laboratory data from patients hospitalized for COVID-19 were collected on a prospective basis. The study was approved by the Institutional Review Board and the Local Ethics Committee (Protocol Number: 8681/1-4-20). Diagnosis of COVID-19 was based on a positive real-time reverse transcriptase polymerase chain reaction of an upper respiratory nasopharyngeal (or oropharyngeal) swab. Disease progression and severity was estimated by maximum FiO_2_% during hospitalization and mortality. We applied the suggested cut-off threshold of 14.5% for RDW and divided patients into high group (RDW ≥ 14.5%) and low group (RDW < 14.5%). The cut-off threshold for RDW in our laboratories was 14.8%. Nonetheless, RDW is calculated based on a standardized technique involving the width of red cells' distribution curve and mean cell size. In particular, it is calculated by dividing the standard deviation of the mean cell size by the mean corpuscular volume of red cells and multiplying by 100 to convert to a percentage. Mann-Whitney test was applied to assess differences in maximum FiO_2_% during hospitalization between high and low RDW group. Kaplan-Meier method was used to analyze survival for patients stratified by RDW on admission. *p*-values < 0.05 were considered statistically significant.

## Results

A total number of 193 hospitalized patients with COVID-19 were enrolled and analyzed (Table 1). Median age was 61 years (95% CI: 58–64). Patients were mostly males (*n* = 139, 72%) and never smokers (*n* = 110, 57%). Thirty eight patients (*n* = 38, 19.7%) required mechanical ventilation at any time during hospitalization. There were no patients under the status— “Do Not Attempt Resuscitation” - in this study group of the first wave of the pandemic. Comorbidities of the study group are summarized in Table 1. All patients were hospitalized and the majority of them received the standard of care at the time of the first wave (azithromycin, hydroxychloroquine, prophylactic dose of low molecular weight heparin). Treatment regimens were relatively homogeneous based on the national protocol. Further details for treatment modalities are presented in Table 2.

**Table 1:** Baseline characteristics.

**Characteristics**	**(*N*, %)**
Total number of patients	193
Age median (%95 CI)	61 (58 to 64)
Males/Females	139/54
Current/ Ex-smokers	83 (43%)
Never smokers	110 (57%)
RDW (%) median (95% CI)	12.8 (95%: 12.6 to 13.1)
Inpatient	193/193 (100%)
Mechanical ventilation	38/193 (19.7%)
Hypertension	62/193 (32.1%)
Diabetes mellitus	17/193 (8.8%)
Cancer	13/193 (6.7%)
Hypothyroidism	9/193 (4.7%)
Atrial fibrillation	8/193 (4.1%)
Heart failure	5/193 (2.6%)
COPD	4/193 (2.1%)
Asthma	2/193 (1.0%)

*COPD, Chronic Obstructive Pulmonary Disease; RDW, Red cell distribution width*.

**Table 2:** Therapeutic compounds administered to study population.

**Characteristics**	**(*N*, %)**
Azithromycin	175/193 (90.7%)
Hydroxychloroquine	170/193 (88.1%)
Low molecular weight heparin (prophylactic dose)	166/193 (86.0%)
Other antibiotics	163/193 (84.4%)
Lopinavir/Ritonavir	33/193 (17.1%)
Colchicine	9/193 (4.7%)
Tocilizumab	5/193 (2.6%)
Remdesivir	3/193 (1.6%)
Anakinra	2/193 (1.0%)

**Figure 1:**
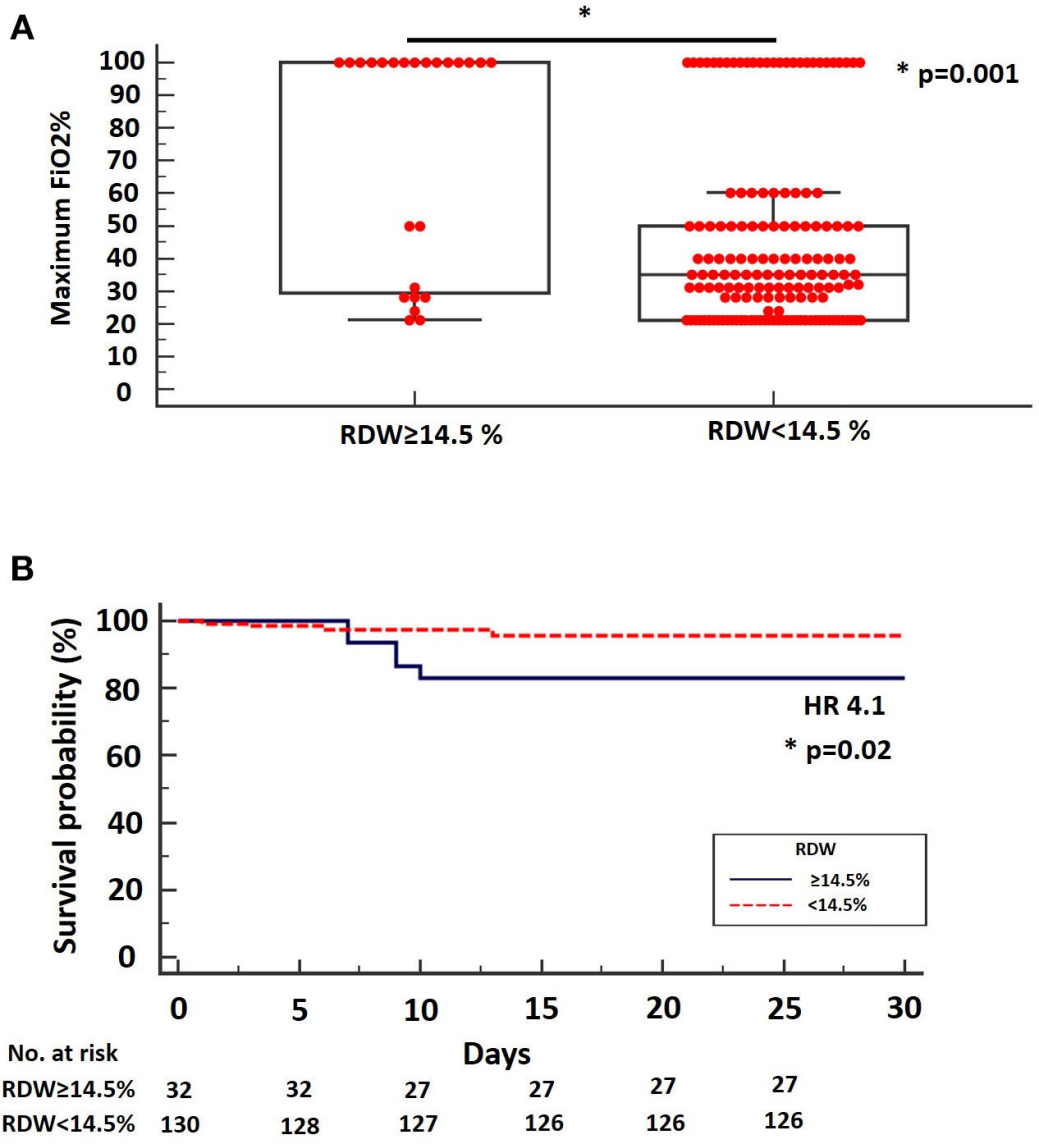
**(A)** Maximum FiO_2_% during hospitalization was significantly higher for patients with SARS-CoV-2 infection and RDW ≥ 14.5% on admission (median: 100, 95% CI: 45.2–100) compared to patients with SARS-CoV-2 infection and RDW < 14.5% on admission (median: 35, 95% CI: 31–40), (*p* = 0.001). **(B)** Kaplan-Meier survival curve using the RDW cut-off threshold of 14.5%. The cut-off threshold of 14.5% differentiated high from low-risk mortality groups. HR = 4.1, 95% CI = 0.88–19.23.

We reported a median RDW on admission of 12.8% (95%: 12.6–13.1). By applying RDW cut-off threshold of 14.5%, as suggested by Foy et al. we managed to identify a subgroup of patients with more advanced disease (high group, *n* = 41, 19.2%) compared to the low group (*n* = 156, 80.8%) as indicated by significant differences in maximum FiO_2_% during hospitalization (median: 100, 95% CI: 45.2–100, vs. 35, 95% CI: 31–40, *p* = 0.0001, respectively) Figure 1A. Interestingly, values of RDW ≥14.5% were strongly associated with increased risk of mortality in the unadjusted analysis (HR: 4.1, 95% CI: 0.88–19.23), (*p* = 0.02), (data for mortality available for 162 patients) (Figure 1B). RDW was not an independent risk factor for mortality when adjusted for age, gender, body mass index and comorbid conditions. However, in the unadjusted analysis, RDW was associated with higher risk of mortality. Finally, RDW could not determine ICU admission in our cohort (data not shown).

## Discussion

Our study demonstrated that baseline RDW is associated both with disease severity and mortality in patients with COVID-19. Our findings exhibit a number of significant attributes that need to be outlined. To our knowledge, this is the first study in the literature that reproduces prognostic accuracy of a previously published threshold of RDW in a completely independent cohort of patients with COVID-19. The reproducibility and concordance of our data support the notion that RDW may serve as a reliable prognosticator and a clinician's friendly biomarker accessible to almost every physician in the world, for patients with COVID-19 (11).

Interestingly, our cohort exhibited lower median RDW levels (12.8%) compared to Foy et al. (survivors: 13.8%, non-survivors:15%) (11). In addition, our cohort exhibited higher percentage of patients (*n* = 156, 80.8%) below the suggested cut-off threshold for RDW (14.5%) compared to Foy et al. (*n* = 1173, 71.5%) (11). These differences might be explained by different stages of disease severity between two cohorts. Given that RDW has been suggested as a biomarker of hypoxia, low RDW on admission in Greece may reflect timely implementation of lockdown and meticulous application of prophylactic measures from patients with chronic lung diseases during the first wave of the pandemic (3.1% of our cohort, *n* = 6) (12). However, exact association of RDW with hypoxia remains to be addressed and elevation of RDW might be associated with alternative pathways. The design of this study cannot lead to definite conclusions. Thus, RDW might instead reflect the severity of lung injury or the underlying comorbid conditions of each cohort.

Despite these attributes, our study exhibited a number of limitations that need to be addressed. We chose maximum FiO_2_ instead of PaO_2_/FiO_2_ due to the fact that PaO_2_/FiO_2_ was not available from all centers. We acknowledge that maximum FiO_2_ required does not directly reflect patients' physiological status, but is also partly dependent on institutional strategy of respiratory support. Moreover, RDW was not an independent risk factor for mortality when adjusted for age, gender, body mass index and comorbid conditions. However, in the unadjusted analysis, RDW was associated with higher risk of mortality. Furthermore, our study was not designed to delineate potential mechanisms underlying RDW increases in the context of COVID-19. SARS-CoV-2 infection has been associated with altered turnover in all white blood cells lineages, as well as with altered platelet dynamics (13). Increases in RDW may not only reflect compensatory upregulation due to excessive blood hypoxemia but also counterregulatory changes of red blood cells turnover due to increased white blood cells and platelet kinetics (14–16). However, the concept that RDW is a non-specific biomarker of general illness and is affected by many confounding factors particularly in the setting of the multi-systemic inflammatory reaction of SARS-CoV-2 infection seems reasonable (11). We believe that a causal -effect relationship between SARS-CoV-2 infection and elevated RDW is highly unlikely. Instead, it is more likely that increased RDW is indicative of patients' comorbidome and thus may identify those patients at increased risk for mortality.

In conclusion, our study provides evidence to support reproducibility and validity of a specified cut-off threshold of RDW as biomarker of disease severity and mortality in two independent cohorts of COVID-19 patients. Further investigation of the exact role of RDW in SARS-CoV-2-infection may reveal mechanisms of potential therapeutic interest. Longitudinal epidemiological studies and population-based analyses are sorely needed to prove these concepts.

## Data Availability Statement

The raw data supporting the conclusions of this article will be made available by the authors, without undue reservation.

## Ethics Statement

The studies involving human participants were reviewed and approved by Institutional Review Board and the Local Ethics Committee (Protocol Number: 8681/1-4-20). The patients/participants provided their written informed consent to participate in this study.

## Author Contributions

TK and AT were involved in study conception, statistical analysis, and drafting of the initial version of the manuscript. KA, OP, VP, and AK were involved in data collection. KA, PB, SL, DB, CG, and AT supervised the work and offered significant intellectual contribution. All authors offered significant intellectual contribution for the last version of the manuscript and approved the final form.

## Conflict of Interest

The authors declare that the research was conducted in the absence of any commercial or financial relationships that could be construed as a potential conflict of interest.

## References

[1] Nathan SD Reffett T Brown AW Fischer CP Shlobin OA Ahmad S. The red cell distribution width as a prognostic indicator in idiopathic pulmonary fibrosis. Chest. (2013) 143:1692–8. 10.1378/chest.12-1368 23238641

[2] Karampitsakos T Dimakou K Papaioannou O Chrysikos S Kaponi M Bouros D. The role of increased red cell distribution width as a negative prognostic marker in patients with COPD. Pulm Pharmacol Ther. (2019) 60:101877. 10.1016/j.pupt.2019.101877 31843703

[3] Karampitsakos T Bouros E Anagnostopoulos A Cholidou K Panou V Korba A Increased red cell distribution width and absolute number of monocytes represent negative prognostic markers in patients with idiopathic pulmonary fibrosis. In: American Thoracic Society 2020 International Conference. Philadelphia, PA (2020). p. A3377 10.1164/ajrccm-conference.2020.201.1_MeetingAbstracts.A3377

[4] Xie J Covassin N Fan Z Singh P Gao W Li G. Association between hypoxemia and mortality in patients with COVID-19. Mayo Clinic Proc. (2020) 95:1138–47. 10.1016/j.mayocp.2020.04.006 32376101 PMC7151468

[5] Tobin MJ Laghi F Jubran A. Why COVID-19 silent hypoxemia is baffling to physicians. Am J Respir Crit Care Med. (2020) 202:356–60. 10.1164/rccm.202006-2157CP 32539537 PMC7397783

[6] Dhont S Derom E Van Braeckel E Depuydt P Lambrecht BN. The pathophysiology of ‘happy’ hypoxemia in COVID-19. Resp. Res. (2020) 21:198. 10.1186/s12931-020-01462-5 32723327 PMC7385717

[7] Wang C Deng R Gou L Fu Z Zhang X Shao F. Preliminary study to identify severe from moderate cases of COVID-19 using combined hematology parameters. Ann Transl Med. (2020) 8:593. 10.21037/atm-20-3391 32566620 PMC7290538

[8] Pan Y Ye G Zeng X Liu G Zeng X Jiang X. Can routine laboratory tests discriminate SARS-CoV-2-infected pneumonia from other causes of community-acquired pneumonia? Clin Transl Med. (2020) 10:161–8. 10.1002/ctm2.23 32508038 PMC7274074

[9] Gong J Ou J Qiu X Jie Y Chen Y Yuan L. A tool for early prediction of severe coronavirus disease 2019 (COVID-19): a multicenter study using the risk nomogram in Wuhan and Guangdong, China. Clin Infect Dis. (2020) 71:833–40. 10.1093/cid/ciaa443 32296824 PMC7184338

[10] Lu G Wang J. Dynamic changes in routine blood parameters of a severe COVID-19 case. Clin Chim Acta. (2020) 508:98–102. 10.1016/j.cca.2020.04.034 32405079 PMC7217800

[11] Foy BH Carlson JCT Reinertsen E Padros I Valls R Pallares Lopez R. Association of red blood cell distribution width with mortality risk in hospitalized adults with SARS-CoV-2 infection. JAMA Netw Open. (2020) 3: e2022058. 10.1001/jamanetworkopen.2020.22058 32965501 PMC7512057

[12] Epstein D Nasser R Mashiach T Azzam ZS Berger G. Increased red cell distribution width: a novel predictor of adverse outcome in patients hospitalized due to acute exacerbation of chronic obstructive pulmonary disease. Respir. Med. (2018) 136:1–7. 10.1016/j.rmed.2018.01.011 29501240

[13] Spiezia L Boscolo A Poletto F Cerruti L Tiberio I Campello E. COVID-19-related severe hypercoagulability in patients admitted to intensive care unit for acute respiratory failure. Thromb Haemost. (2020) 120:998–1000. 10.1055/s-0040-1710018 32316063 PMC7295272

[14] Higgins JM Mahadevan L. Physiological and pathological population dynamics of circulating human red blood cells. Proc Natl Acad Sci USA. (2010) 107:20587–92. 10.1073/pnas.1012747107 21059904 PMC2996693

[15] Patel HH Patel HR Higgins JM. Modulation of red blood cell population dynamics is a fundamental homeostatic response to disease. Am J Hematol. (2015) 90:422–8. 10.1002/ajh.23982 25691355 PMC4717489

[16] Chaudhury A Miller GD Eichner D Higgins JM. Single-cell modeling of routine clinical blood tests reveals transient dynamics of human response to blood loss. Elife. (2019) 8:e48590. 10.7554/eLife.48590 31845889 PMC6917488

